# Effect of Gas Type and Its Pressure on Nanobubble Generation

**DOI:** 10.3389/fchem.2021.630074

**Published:** 2021-03-25

**Authors:** Nikolai F. Bunkin, Alexey V. Shkirin, Nikita V. Penkov, Mikhail V. Goltayev, Pavel S. Ignatiev, Sergey V. Gudkov, Andrey Yu. Izmailov

**Affiliations:** ^1^ Bauman Moscow State Technical University, Moscow, Russia; ^2^ Prokhorov General Physics Institute of the Russian Academy of Sciences, Moscow, Russia; ^3^ National Research Nuclear University MEPhI, Moscow, Russia; ^4^ Federal Research Center “Pushchino Scientific Center for Biological Research of the Russian Academy of Sciences”, Institute of Cell Biophysics of the Russian Academy of Sciences, Moscow, Russia; ^5^ JSC “Production Association “Ural Optical and Mechanical Plant named after E.S. Yalamov” (UOMZ), Ekaterinburg, Russia; ^6^ Federal State Budgetary Scientific Institution “Federal Scientific Agroengineering Center VIM”(FSAC VIM), Moscow, Russia

**Keywords:** gas nanobubbles, bubstons, optical breakdown, dynamic light scattering, laser phase microscopy, stabilization of nanobubbles

## Abstract

The dependence of the volume number density of ion-stabilized gas nanobubbles (bubstons) on the type of gas and the pressure created by this gas in deionized water and saline solution has been investigated. The range of external pressures from the saturated water vapor (17 Torr) to 5 atm was studied. It turned out that the growth rate of the volume number density of bubstons is controlled by the magnitude of the molecular polarizability of dissolved gases. The highest densities of bubstons were obtained for gases whose molecules have a dipole moment. At fixed external pressure and the polarizability of gas molecules, the addition of external ions leads to a sharp increase in the content of bubstons.

## Introduction

Recently, there has been an increased interest in nanobubbles (gas bubbles with a size of 100–200 nm) in the volume of a liquid, see review (Alheshibri et al., 2016) and the references therein. The interest in these objects is primarily due to a wide range of their applications, mainly in medicine and adjacent areas. Nanobubbles are an interdisciplinary topic that connects physics, chemistry, life sciences, and engineering. Currently, new technologies related to the use of nanobubbles are rapidly developing. We note right away that the size 100–200 nm refers rather to the submicron than to the nanoscale. Therefore, it is necessary to clarify what we mean by a nanobubble. The authors of the study (Agarwal et al., 2011) define nanobubbles as bubbles with a diameter of less than 200 nm. However, they also define microbubbles as bubbles with a diameter in the range of 10–50 μm, which leaves a huge gap between the upper boundary of nanobubbles (200 nm) and the lower boundary of microbubbles (10 µm). The authors of the study (Wu et al., 2012) use the term “submicron sized bubbles” as a synonym for “nanobubbles” to describe bubbles several hundred nanometres in diameter. At the same time, in (Cho et al., 2005) the authors use the term nanobubbles for bubbles with a diameter of less than 1000 nm (1 μm). Tsuge in his monography (Tsuge, 2015) argued that for bubbles to be considered nanobubbles, they must have a diameter of less than 100 nm, but the author acknowledged the fact that most researchers set a 1 μm boundary for the diameter of nanobubbles. It is also worth noting that in accordance with ISO/TS 80004-2:2015, the nano prefix used in nanotechnologies must be reserved for objects with at least one characteristic length less than 100 nm. In the case of nanobubbles, the objects are always spherical thus characteristic size is always the diameter. In a number of recent works devoted to nanobubbles [see, for example (Köse et al., 2020)], it is generally accepted that nanobubbles are gas particles with a gas core diameter of less than 1 μm, while for microbubbles this diameter is in the range of 1–10 µm. In the future, we will adhere to this particular classification.

Since nanobubbles are compressed by enormous surface tension forces, the question of their stability always arises. Epstein and Plesset studied the dynamic evolution of bubbles (Epstein and Plesset, 1950) and indicated that bubbles in solution will shrink or expand depending on whether the dissolved gas in the solution is supersaturated or not. The time required for complete bubble dissolution can be determined using the Epstein and Plesset (E-P) equation (Yasui, 2015), which predicts that the survival time for nanosized bubbles should be less than 0.02 s. In contrast to both thermodynamic and kinetic arguments indicating the instability of bulk nanobubbles, numerous experimental works reported on the existence of bulk nanobubbles (Thorpe et al., 1982; Ohgaki et al., 2010; Ushikubo et al., 2010; Oh and Kim, 2017). More importantly, the observed nanobubbles have a lifetime of several minutes to weeks, which is significantly longer than the theoretical prediction. Various hypotheses have been proposed for the interpretation of unexpected observations (Seddon et al., 2012; Nirmalkar et al., 2018; Yasui et al., 2018), although there has not yet been a single conclusion (Häbich et al., 2010; Sedlák and Rak, 2013; Kononov, 2015). Below we give a brief overview of our current understanding of the unexpected stability of bulk nanobubbles.

Several mechanisms have been proposed for stabilizing gas nanobubbles, but for most of them there is experimental evidence to the contrary. We should mention here four main mechanisms for nanobubble stability: the contaminant mechanism (Jin et al., 2007; Sedlák and Rak, 2014; Nirmalkar et al., 2019), the “skin” mechanism (Fox and Herzfeld, 1954; Strasberg, 1959; Ohgaki et al., 2010; Weijs et al., 2012; Zhang et al., 2016), the surface zeta potential mechanism (Ushikubo et al., 2010; Oh and Kim, 2017; Millare and Basilia, 2018) and the high-density mechanism (Huang et al., 2013; Zhang et al., 2013). We however will not describe these mechanisms in detail here.

An alternative mechanism for stabilizing nanobubbles in the bulk of liquid free from foreign solid impurities is the adsorption of ions of the same sign (cations or anions) on the inner surface of nanobubbles. Owing to ionic adsorption, nanobubbles acquire a surface electric charge, and the stabilization is realized due to a balance between surface tension forces and negative electrostatic pressure caused by the repulsion of adsorbed ions. Such nanobubbles are called bubstons (abbreviation for *bubbles stabilized by ions*). Since the surface tension for bubstons is compensated by the negative electrostatic pressure of adsorbed ions, the gas pressure inside the bubstons is equal to atmospheric, i.e., bubstons are stable both mechanically and diffusionally. Theoretical aspects of the stabilization of bubstons are presented in (Bunkin and Bunkin, 2016); the bubstons themselves were first mentioned in (Bunkin and Bunkin, 1992). Papers (Bunkin et al., 2012; Bunkin et al., 2016; Yurchenko et al., 2016) are devoted to the experimental study of bubstons using various laser techniques.

This work is devoted to the study of the effect of pressure and type of gas on the nucleation process of bubstons. Note that nucleation of the bubble phase occurs when the liquid is supersaturated with a dissolved gas. Experimental works on a topic related to the study of supersaturation state, were started in the seventies of the last century, see (Hemmingsen, 1975). In this work, it was initially assumed that there are no long-lived nucleation centers in the liquid. At the same time, it was shown that exerting high pressure of various gases to water can significantly increase the supersaturation threshold for cavitation. This was interpreted as a consequence of the pre-existing small cavities (cavitation nuclei): the high pressure increases the solubility of the gas in water and thus forces the gas confined in small cavities to dissolve. The maximum supersaturation tension without cavitation, which is the minimum possible limit of spontaneous bubble formation, was 140 atm for O_2_, and Ar, 190 atm for N_2_ and 300 atm for He, while massive cavitation occurred at higher supersaturations by 20–30 atm. It is interesting that in this work, in the case of helium, the regime of massive cavitation was not obtained at all, which points to the peculiarities of the nucleation regime of nuclei of the nanobubble phase for helium. At the same time, helium-containing nanobubbles were observed on the surface of a hydrophobized silicon substrate, see (Van Limbeek and Seddon, 2011). As was shown in this work, gas type is a key parameter in the nucleation of surface nanobubbles; all experiments were carried out under equilibrium conditions, i.e., far from the supersaturation state. Since the surface of the substrate was hydrophobized and, apparently, was not absolutely smooth, the nucleation of nanobubbles arose on local roughness (spikes, crevices, and cracks) of the hydrophobic substrate, i.e., in this case, we are dealing with the stabilization of nanobubbles due to the contaminant mechanism. It turned out that there exists an optimal temperature for nanobubble nucleation between ∼35 and ∼40°C that appears to be weakly dependent on gas type. Surprisingly, nanobubble nucleation does not directly depend either on the solubility of a specific gas in water or the relative adsorption strength of the gas to the substrate. This indicates that nanobubbles do not form solely because of the amount of gas available in the bulk.

Experiments related to the realization of a supersaturated state of a dissolved gas in water are rather complicated, while the measurements of the bubble nucleation threshold require cumbersome saturation at high pressures. In (Rubin and Noyes, 1987), the method of chemical reactions at ambient temperature and normal pressure was used instead to obtain supersaturated solutions. The degree of supersaturation is then measured as the amount of gas released when rapid stirring or sonication is suddenly initiated; according to (Rubin and Noyes, 1987), sonication/stirring increased the rate of chemical reactions. The threshold for nucleation is the limit beyond which it is impossible to reach the supersaturation level. The nucleation thresholds for the diatomic gases H_2_, N_2_, O_2_, CO, and NO in aqueous solutions lie between 0.012 and 0.07 M, while for CO_2_ it is at least 0.4 M. Note that the absence of long-lived nucleation centers of nanobubbles was assumed in (Rubin and Noyes, 1987). The fact that the generation of bubbles requires not only supersaturation with dissolved gas, but also the application of an external force as sonication/stirring, indicates that this force appears to lead to the formation of nanometer cavities, which are filled with dissolved gas, and thus, the supersaturation state is effectively depleted.

It is necessary to mention works in which supersaturation is created in liquids, where nuclei of the nanobubble phase are obviously present. For example, in recent work (Azevedo et al., 2016) a ‘‘new” technique was developed to generate highly loaded dispersions of nanobubbles about 300 nm in size after depressurizing air that saturated water at low pressures (<3 bar) and decreasing the air/water surface tension to approximately 49 mN/m. As a result, a high density of aqueous bulk nanobubbles was created (1.6⋅10^9^ NBs mL/1), which lasted for at least 2 weeks. We should also refer to works (Jin et al., 2019; Wang et al., 2019), in which the efficient generation of nanobubbles was obtained by periodical pressurization - depressurization of water sample in a sealed cell with the help of different gases: the authors of (Wang et al., 2019) investigated N_2_, O_2_, and CO_2_, while in (Jin et al., 2019) the hydrophobic gas SF_6_ was studied. In (Wang et al., 2019), in particular, the technique of freeze-fracture transmission electron microscope [see, e.g., (Uchida, et al., 2011)] was applied to study the nanobubbles arising due to supersaturation. In works (Azevedo et al., 2016; Jin et al., 2019; Wang et al., 2019), it was found that the volume number density of nanobubbles in a supersaturated state is significantly higher than that for water under normal conditions. The effects of gas concentration and species on the coalescence and growth of nanobubbles were systematically investigated in theoretical study (Li et al., 2018), where, using molecular dynamics simulations, it was shown that with increasing gas concentration, not only surface nanobubbles but also bulk nanobubbles are formed.

Apparently, the most reliable data on the nucleation threshold of nanobubbles in solutions supersaturated with various dissolved gases and free from long-lived nucleation centers, can be obtained in electrophoresis experiments with the use of platinum nanoelectrodes. For example, in (Chen et al., 2014) the critical surface concentration of dissolved H_2_ required for nanobubble nucleation was measured to be ∼ 0.25 M; this value is ∼ 310 times greater than the saturation concentration at room temperature and pressure and does not depend on the nanoelectrode size. In (Chen et al., 2015), the critical surface concentration of dissolved N_2_ was measured to be ∼0.11 M, which is ∼160 times higher than the N_2_ saturation concentration at room temperature and atmospheric pressure. The results of this work suggest that the size of stable gas bubble nuclei depends only on the local concentration of N_2_ near the electrode surface. Similar results were obtained in (Ren et al., 2017) for oxygen: a single bubble nucleated when the concentration of dissolved O_2_ on the surface of the Pt electrode reaches ∼0.17 M. This nucleation concentration is ∼130 times higher than the equilibrium saturation concentration of O_2_ and is independent of the electrode size. Finally, in (Ren et al., 2020), the controlled formation of individual CO_2_ nanobubbles on Pt nanoelectrodes was reported. It turned out that CO_2_ bubbles nucleate when the concentration of CO_2_ at the Pt electrode was exceeds ∼ 0.6 M, which means 18-fold supersaturation.

Summarizing this section, we conclude that the nucleation of gas nanobubbles depends on the type of gas molecules. Furthermore, there is nothing unusual in the fact that the nucleation of nanobubbles must occur should conditions of supersaturation of the liquid with a dissolved gas. However, the question of the mechanism of nanobubble nucleation under normal conditions (i.e., far from the supersaturation point) remains open and requires special consideration. If we assume that the nucleation centers of bubstons are gas molecules dissolved in a liquid then it would seem that the volume number density of nanobubbles should be determined by the partial pressure of a given gas above the liquid surface and the solubility of this gas in the liquid. It should be noted that for atmospheric air under normal conditions the volume number density of molecules of dissolved gas in water can be estimated as *n*_*g*_ = *L*⋅*R*, where *L* = 2.7⋅10^19^ cm^−3^ is the Loschmidt number, *R* ∼ 10^−2^ is the solubility constant [Henry's constant, see, e.g., (Sander, 2015)], i.e., *n*_*g*_ ∼ 10^17^ cm^−3^ << *n*_*l*_ = 3.3⋅10^22^ cm^−3^, where *n*_*l*_ is the number density of water molecules.

Note that, as shown in (Bunkin et al., 2012; Bunkin et al., 2016; Yurchenko et al., 2016), the volume number density of bubstons grows with an increase in the content of dissolved ions. Thus, when considering the mechanism of nanobubble nucleation, we must take into account the concentration and, possibly, the specific properties of dissolved ions in relation to their hydration ability; here, we are talking about the so-called cosmotropic (structure-making) and chaotropic (structure-breaking) cations and anions, see, for example (Ninham et al., 2011; Duignan et al., 2014a; Duignan et al., 2014b), and references therein. Thus, when studying the nucleation mechanism of nanobubbles, it is necessary to take into account, first, the local interaction of gas molecules with the molecular environment of water, and, second, the interaction of gas molecules with ions, taking into account their chaotropic/cosmotropic properties. In our previous work (Yurchenko et al., 2016) the ion-specific effects in the stabilization of bubstons were theoretically and experimentally studied for univalent and divalent anions and cations. Concluding this section, it is pertinent to note that if we are dealing with an ionic solution, then, as shown in our theoretical work (Bunkin and Shkirin, 2012) the effect of clustering of bubstons is possible: dimers and more intricate complexes with fractal properties can be formed, see our works (Bunkin et al., 2012; Bunkin and Shkirin, 2012) for more details.

## Theoretical Section

### Mechanism of The Nucleation of Nanobubbles

The question of nucleation of nanobubbles can be reformulated as follows: how can mesoscopic cavities arise in water far from the boiling point? Since in this work, in particular, the bubston phase in physiological saline solution (0.14 M NaCl) will be studied, we assume that we are dealing with an equilibrium solution of Na^+^ and Cl^−^ ions, in which gas molecules are dissolved. The process of the formation of bubston nuclei is identical to the process of the nucleation of ionic crystals of NaCl salt in aqueous solutions of Na^+^ and Cl^−^ ions. The crystallization of NaCl starts with the formation of droplets of ionic condensate, i.e., ionic crystals of NaCl with mesoscopic (nanometer) sizes. Having arisen with some probability, a droplet of ionic condensate can remain quasi-stable only at a sufficiently high concentration of dissolved Na^+^ and Cl^−^ ions. In supersaturated solutions, further growth of such a droplet results in the formation of macroscopic NaCl crystals. In solutions far from saturation, such droplets cannot remain stable; these droplets eventually decay, which has the character of a “Coulomb explosion.” This leads to the formation of mesoscopic cavities with radius *r*_o_. Below, the mechanism of the formation of a mesoscopic droplet of ionic condensate will be considered at a qualitative level.

First of all, it is necessary to answer the question of where the growth of ionic droplets starts from. We assume that there are no stable inhomogeneities in the solution (for example, in the form of foreign solid particles) and, therefore, the only “defects” in the structure of an aqueous ionic solution are neutral gas particles. Ionic droplets begin to grow on such defects as a result of the diffusion of dissolved ions onto the droplet surface. The first stage is the formation of “gas particle + ion” complexes (which we will further call “ionic dimers”), arising from the adhesion of ions to neutral gas particles. The energy of the affinity of ions and dissolved gas molecules is electrostatic and is given by the formula $U=\beta e^{2}/2{\left(\delta_{g}+\delta_{i}\right)}^{4},$ where *β* is the electronic polarizability of gas molecules, *δ*_*g*_ is their radius, and *δ*_*i*_ is the ion radius. If the lifetime of such dimers *τ*_*d*_ is long enough, then condensation of these ions can occur as a result of the diffusion of surrounding ions onto the “surface” of such dimer, and droplets of ionic condensate are formed with some probability. The ions have a spatial arrangement in the form of a simple cubic lattice, i.e., the distance between the nearest ions of opposite signs is $a=\left(\delta_{Na^{+}}+\delta_{Cl^{-}}\right)=2.8$Å, which is approximately equal to half the lattice constant. As is known, such an arrangement of ions leads to the fact that the Coulomb attraction between ions of opposite sign is stronger than the repulsion between ions of the same sign. In other words, this means that all ions in the droplet are located in sufficiently deep potential wells, so that such a droplet is mechanically stable.

Direct calculation of the Coulomb interaction of one ion of a droplet with other ions shows that the smallest number of condensed ions of both signs, at which the maximum mechanical stability is achieved, is 27. In this case, 14 ions of the same sign occupy 6 vacancies of the first coordination sphere (its radius is equal to *a*), and 8 vacancies of the third sphere (radius is $\sqrt{3}a$), 13 ions of opposite sign fill 12 vacancies of the second sphere (radius is $\sqrt{2}a$), and one ion is located on the initial ionic dimer. With such a “three-layer” structure of the ionic droplet, the interaction energies of one ion with other 26 ions of the droplet are approximately equal to the following values: on the first sphere, the ion energy is equal to $w_{1}=-1.3\cdot e^{2}/a$; on the second sphere, we have *w*_2_ = −2.0⋅*e* ^2^ */a*; on the third sphere $w_{3}=-1.0\cdot e^{2}/a,$ and the energy of the ion in the position of the initial dimer is *w*_0_ = −2.1⋅*e* ^2^ */a*. The droplet has the shape of a cube with length of 2*а* = 5.6 Å. This droplet consisting of 27 ions can be called a “dry droplet”; here, we emphasize the fact that an absolutely stable state takes place only outside the solution, i.e., when there is no interaction of the ions with water molecules. In an aqueous solution, there is a significant decrease (in absolute value) in the binding energy between the ions due to the polarization of the water molecules surrounding the ion droplet, i.e., the depth of potential wells (in which the ions are located) decreases. It is clear that the ions located on the third coordination sphere are subject to the most significant decrease in the binding energy.

A simple estimate based on the Gauss electrostatic theorem shows that for these ions the binding energy $\left|w_{3}\right|$ decreases by a factor of (εΔΩ/4π) (here *ε* = 80 is the dielectric permittivity of water, and ΔΩ=4π−π/2=7π/2 is the solid angle, at which the water molecule is visible from the point on the third sphere, where the ion is located). Thus we obtain that the binding energy $\left|w_{3}\right|$ decreases by about 70 times and becomes equal to $\left|w_{3}^{'}\right|=e^{2}/70\cdot a=$ 0.073 eV, while $\left|w_{3}^{'}\right|/kT=$2.8. The binding energies of the remaining ions of the droplet are also subject to a significant decrease. As a result, the ionic bond in the droplet due to thermal processes loses its stability, which leads to destroying the droplet and, thus, to the formation of a mesoscopic empty cavity with a radius of *r*_o_ **≈** *а* ≈ 3 Å; it can be shown that this result is independent of external pressure up to 10^3^ atm. Note that in (Hemmingsen, 1975), the estimates of the sizes of the initial critical vapor nuclei radius at atmospheric hydrostatic pressure were made at a qualitative level: in accordance with the results of (Hemmingsen, 1975), the radius of the initial stable vapor-gas bubble should be less than 10^−7^ cm. Thus, our estimate is in a qualitative agreement with the results of (Hemmingsen, 1975).

Apparently, a necessary condition for the mesoscopic void formation is that the lifetime $\tau_{d}$ of the ionic dimers should be sufficiently long, and during this time the dimers can transform to an ion droplet with a radius of *а* = 2.8 Å. Here are some quantitative estimates. The time $\tau_{d}=\nu_{d}^{-1}\exp\left(U/kT\right),$ where *ν*_*d*_ is the vibration frequency of the dimer, *U* is the energy of the affinity of ions and gas particles, *k* is the Boltzmann constant, and *T* is the absolute temperature. According to the well-known formula of classical mechanics$\nu_{d}=\left(1/2\pi \right)\sqrt{w^{\prime \prime }\left(0\right)/\mu },$ where $w\left(x\right)=\beta e^{2}/2{\left(\delta_{0}+x\right)}^{4}\text{​},\delta_{0}=\delta_{g}+\delta_{i},\mu$ is the reduced mass of a gas particle and an ion, $w"\left(0\right)=10\beta e^{2}/\delta_{0}^{6}$ is the "spring stiffness" due to the Coulomb interaction between an ion and a gas molecule, $w\left(0\right)=U=\beta e^{2}/2\delta_{0}^{4},$ and thus the ion dimer lifetime is given by formula

$$\tau_{d}=\sqrt{\frac{4\mu \delta_{0}^{6}}{\beta e^{2}}}\exp\left(\frac{\beta e^{2}}{2\delta_{0}^{4}kT}\right).$$ (1)

It is of interest to estimate the time $\tau_{d}$ for dimers (N_2_ + Na^+^) and (N_2_ + Cl^−^) for the case when the ionic aqueous solution is in equilibrium with nitrogen (air). For the dimer (N_2_ + Na^+^) we have *β* = 1.76⋅10^−24^ cm^−3^, *δ*_0_ = 1.58 + 0.98 = 2.56 Å, *μ* = 2.1⋅10^−23^ g, and formula (1) gives $\tau_{d}=$2.4⋅10^−8^ s. For the dimer (N_2_ + Cl^−^) we have *δ*_0_ = 1.58 + 1.81 = 3.39 Å, *μ* = 2.6⋅10^−23^ g, and $\tau_{d}=$2.6⋅10^−11^ s. Thus, the lifetime of the (N_2_ + Na^+^) dimer is four orders of magnitude longer than that of the (N_2_ + Cl^−^) dimer.

The characteristic time of the formation of an ion droplet due to diffusion of the surrounding ions onto the ionic dimer is $\tau_{d}r=a^{2}/D_{i}^{eff},$ where $D_{i}^{eff}$ is effective diffusion coefficient, significantly different from diffusion coefficients for Na^+^ and Cl^−^ ions (which at *t* = 25°C are equal to 1.36⋅10^−5^ cm^2^/s and 2.0⋅10^−5^ cm^2^/s, respectively). The physical meaning of the coefficient $D_{i}^{eff}$ is that it stands only for the diffusion flux $D_{i}^{eff}\cdot \nabla n_{i}$ of Na^+^ and Cl^−^ions onto the surface of a growing ion droplet, which allows this droplet to remain stable, i.e., when the Na^+^ and Cl^−^ions fit into a simple cubic lattice. The ionic flows that do not meet this condition are excluded, since such flows result in the destruction of the droplet.

We will assume $D_{i}^{eff}$ equal to $\alpha \left(D_{Na^{+}}+D_{Cl^{-}}\right)/2,$ where *α* << 1 and has the meaning of the probability that the droplet grows to size *a*, while remaining stable. We obtain $\tau_{d}r=a^{2}/\alpha \left(D_{Na^{+}}+D_{Cl^{-}}\right)$ ≈ (5⋅10^−11^ */α*) sec (where *a* = 2.8 Å), and, accordingly, the necessary condition for the formation of a mesoscopic cavity takes the form $\tau_{d}>\tau_{dr}=\left(5\cdot 10^{-11}/\alpha \right)$ sec. Since *α* << 1, the lifetime of the dimers must satisfy the condition $\tau_{d}>>5\cdot 10^{-11}$ sec. The estimates of the times *τ*_*d*_ show that this condition can be fulfilled only if the droplet grows on the dimer (N_2_ + Na^+^), for which $\tau_{d}=$2.4⋅10^−8^ sec. The probability *α* in this case must exceed 2⋅10^−3^; this means that at least two dimers (N_2_ + Na^+^) out of a thousand should grow to the size of a droplet with radius *a*. The question of whether such values of the probability *α* can be realized is still open, and therefore, of course, we cannot assume that the formation of mesoscopic cavities occurs only by the above mechanism. Summarizing, in aqueous NaCl solutions, bubston nuclei are most likely formed as a result of occurring ionic dimers (N_2_ + Na^+^).

At the same time, to stabilize a bubston, adsorption of ions on its inner surface is necessary, see (Bunkin and Bunkin, 1992; Bunkin et al., 2012; Bunkin and Bunkin, 2016; Bunkin et al., 2016; Yurchenko et al., 2016). As shown in (Yurchenko et al., 2016), it is the chaotropic anions that are adsorbed on the inner surface; in NaCl solution, bubston is stabilized due to the adsorption of Cl^−^ anions, whereas its nucleation, as shown above, is due ionic dimers (N_2_ + Na^+^). Note that, according to the results of (Kelsall et al., 1996), in pure (free of external ionic impurities) water, the “liquid - gas” intersurface acquires a charge due to the adsorption of ОН^−^ ions; in this case the stabilization of bubstons is due to the adsorption of these anions.

The mesoscopic cavities (mesocavities) described above will be called viable bubston nuclei. Thus, the rate of specific (per unit volume) generation of bubstons with volume number density *n*_*b*_ is defined by the obvious formula

$$\frac{\delta n_{b}}{\delta t}\propto d_{eff}\cdot D_{i}n_{i}n_{g}^{s}\exp\left(-\Delta \varphi /kT\right),$$ (2)

where *n*_*i*_ is the density of dissolved ions, *D*_*i*_ is their diffusion coefficient, Δ*φ* is the minimum increment in the thermodynamic potential of a solution that is realized during the formation of a single mesocavity. The coefficient *d*_*eff*_, which has the dimension of length, reflects the effective dependence of the rate *δn*_*b*_ */δt* on the radii of ions and gas-hydrate inhomogeneities Λ (molecular complexes consisting of gas molecules and water molecules, see below). It is the scale *d*_*eff*_ that should be considered as the effective size of the inhomogeneities. The calculation shows that for water at pressures up to 10^3^ atm. The increment Δ*φ* is about 1 eV. After their emergence, the nuclei (mesocavities) grow and eventually become stable. The stability state corresponds to bubstons with a radius of *R* ≈ 100–200 nm. The formation of bubstons occurs in the process of diffusion on their surface of ions capable of adsorption and filling the expanding cavity with a dissolved gas. When the specific resistance of water is 10 MΩ⋅cm, the ions have a volume number density *n*_*i*_ ≈ 3⋅10^14^ cm^−3^. It is obvious that the density *n*_*b*_ of bubstons increases with increasing the volume number density of dissolved gas molecules $n_{g}^{s}$. Thus, by changing the content of dissolved gas, it becomes possible to vary the value of *n*_*b*_, and degassing of the liquid sample ($n_{g}^{s}\to 0$) makes it possible to completely remove bubstons.

It is clear that formula (2) does not take into account the interaction between a gas molecule and the water dipoles surrounding this molecule. As is known, gas molecules can have a dipole moment (for example, NO and CO) or, in the absence of their own dipole moment, can be polarized in the dipole environment of water molecules. Thus, the interaction of a gas molecule with surrounding water molecules can be considered in the approximation of a dipole-dipole interaction. Thus, a potential well for the dissolved gas molecule is formed. Obviously, the depth of this well is expressed as

$$U_{0}∼-\frac{\left(p_{1}p_{2}\right)}{\Lambda^{3}},$$ (3)

where (**р**_1_ **р**_2_) is the scalar product of the effective dipole moment **р**_1_, formed by the water molecules surrounding the gas particle, and the dipole moment **р**_2_ = *β* **Е** of the gas molecule, where *β* is the electronic polarizability of the gas molecule, Λ is the distance between the centers of the dipoles **р**_1_ and **р**_2_. A gas molecule surrounded by water dipoles is affected by a fluctuating electric field **E**; these fluctuations are obviously associated with the vibrational-librational dynamics of water molecules. It is obvious that the effective angle between the dipole moments **р**_1_ and **р**_2_ also fluctuates, i.e., the value of *U*_0_ also changes, and if at a certain moment of time the condition *U*_0_ < *kT* is met, and then the gas molecule is no longer captured by this potential well. This, incidentally, explains the fact that for most gases, the solubility in water decreases with increasing temperature. For water and nitrogen molecules, we have $\Lambda =\delta_{H2O}+\delta_{N2},$ where δ_N2_ = 1.58 Å, *δ*_Н2О_ = 1.38 Å are the radii of nitrogen and water molecules, $p_{2}=p_{N2}=\beta_{N2}E$ is the dipole moment of a nitrogen molecule in the field **E** induced by water molecules surrounding the N_2_ molecule with an effective dipole moment **р**_1_, *β*_N2_ is the electronic polarizability of nitrogen molecules. Unfortunately, further estimates of the value *U*_0_ are not possible, since we do not know the values of **р**_1_ and, accordingly, **E**.

Obviously, if the rate *δn*_*b*_ */δt* of bubston generation depends not only on the content of the dissolved gas and the concentration of ions, but also on the specific interaction between a gas molecule and a dipole environment (water molecules), then formula (2) should be refined. The aim of this work is to experimentally verify the mechanism of bubston nucleation depending on the pressure and the type of dissolved gas (i.e., in fact, on the polarizability/dipole moment of gas particles) for pure (deionized) water and a saline solution (aqueous NaCl solution, the ion concentration *C* = 0.14 M). Here, we study the effect of pressure on the generation of bubstons in a wide range: from 17 Torr (saturated vapor pressure of water at room temperature) to 5 Atm.

## Experimental Techniques

### Optical Breakdown

**FIGURE 1:**
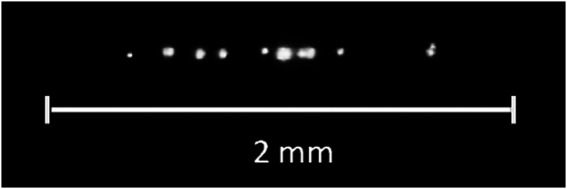
Pattern of optical breakdown in water irradiated with a laser pulse at a wavelength of *λ* = 1,064 nm, the pulse duration is 15 ns.

As shown in (Bunkin and Bunkin, 1993), bubstons serve as centers of optical (induced by laser radiation) breakdown in water. Optical breakdown [see, e.g., a comprehensive review (Vanraes and Bogaerts, 2018)] is the appearance of a plasma spark at the focus of a laser beam, the threshold of which (in terms of radiation intensity) is two to three orders of magnitude lower than the value corresponding to laser-induced multiphoton ionization of water molecules. Breakdown occurs as a result of the development of an electron avalanche inside individual bubstons, which occur in the lens caustic during a laser pulse, and manifests itself in the formation of local plasma flashes, see Figure 1.

It should be noted that the optical breakdown is sporadic; at fixed parameters of the laser pulse, breakdown occurs only with a certain probability *W*. This unambiguously indicates that the breakdown is initiated by some objects that accidentally fall into the focal region with a volume *V* determined by the focusing conditions. The probability of finding a bubston inside such a volume is determined by the average volume number density of bubstons *n*_*b*_ and the volume *V*. According to the Poisson distribution, we have $P\left(N\right)=\exp\left(-n_{b}V\right)\frac{{\left(n_{b}V\right)}^{N}}{N!},$ where *N* is the number of bubstons inside the volume *V* during the laser pulse, *n*_*b*_ *V* is the average number of bubstons in the volume *V*. The probability of finding *N* ≥1 bubstons in the volume *V* is given by the formula

$$W=\;\sum_{N=1}^{\infty }P\left(N\right)=1-P\left(0\right)=1–\exp\left(-n_{b}V\right).$$ (4)

The experimentally measured breakdown probability is determined by the formula *W*_exp_ *= N*_1_ */N*_o_, where *N*_1_ is the number of breakdown flashes for a total fixed number *N*_o_ of laser shots. For *n*_*b*_ *V* << 1 we have

$$W_{\exp}=W\approx n_{b}V\approx N_{1}/N_{0},$$ (5)

and thus the experimentally measured probability (*N*_1_/*N*_o_) allows us to find with a certain accuracy the volume number density of bubstons

$$n_{b}\approx \left(N_{1}/N_{0}\right)\cdot V^{-1}=W_{\exp}\cdot V^{-1}.$$ (6)

The radiation of a single-mode repetitively pulsed Nd^3+^:YAG laser (wavelength *λ* = 1064 nm, pulse duration 15 ns at half-height of the pulse, pulse repetition rate 2 Hz) was directed using a telescope and a lens system into a cell with a liquid sample, where an optical breakdown was excited. The pulse energy was varied in the range of 0.5–1 mJ. Breakdown occurred at a depth of 5 mm with respect to the liquid surface. The radiation intensity in the waist was 4.7⋅10^9^–9.4⋅10^9^ W/cm^2^; this significantly exceeds the theoretical value of the breakdown threshold inside bubstons at this wavelength, see the corresponding estimates in (Bunkin and Bunkin, 1993). In our case, the focusing of radiation was rather tight: the caustic length was *L*_F_ = 1.7 mm, the beam radius in the waist was *R*_o_ = 15 μm, i.e., we have for the beam cross section in the waist *S*_o_ = 7.1·10^−6^ cm^2^.

Since the radiation at this wavelength belongs to the near-IR range, and the spectrum of the plasma flash belongs to the visible and near UV ranges, laser radiation did not obscure the visual observation of the breakdown. Breakdown flashes (their number was equal to *N*_1_) were recorded using a photoelectron cathode with subsequent computer processing. The breakdown volume (volume of the focal region *V*) represents two cones, directed towards each other with a base area *S*_o_ and a height of 0.5*L*_F_, i.e., *V* ≈ 0.4⋅10^−6^cm^3^. The equilibrium density of bubstons in deionized water is *n*_*b*_∼ 10^5^–10^6^ cm^−3^ [see the estimate in (Bunkin et al., 2016) and also Figure 5 below], so we can assume the relation *n*_*b*_ *V* << 1 is satisfied. Substitution into Eq. 5 gives that at least one bubston falls into the volume *V* with the probability *W*_exp_ ≤ 0.4, i.e., breakdown will be sporadic. Below we also present the results of the measurements of the volume number density of bubstons using dynamic light scattering (DLS); however, at *n*_*b*_ < 10^6^ cm^−3^ the DLS technique is no longer effective.

We measured the breakdown probability with decreasing dissolved gas content. The experiments were carried out as follows. A cylindrical cell with a volume of 150 ml and a radius of 40 mm, sealed with a Teflon vacuum valve, was half filled with water produced by a Milli-Q setup (resistivity 7 MΩ⋅cm, pH = 5.5) and saturated with atmospheric air; the volume free from water we will term “free volume.” Next, the liquid sample was subjected to a degassing procedure, which took place in several stages; each stage is numbered *N* = 1, 2, etc. At the first stage, the free volume of the cell, filled with air at a pressure of *p* = 1 atm, was connected to an oil-free foreline pump for 1 min, and a pressure of P = 10^−3^ Torr was created in the free volume. Then the cell was disconnected from the foreline pump and sealed. After that, the liquid was settled for a day; during this time, the free volume was filled with equilibrium water vapor and dissolved gas molecules that left the liquid. At the end of the day, the pressure *P* in the free volume and the probability of breakdown *W*_exp_ were measured again. The measurement of these values means the end of the first stage (*N* = 1). The second and subsequent stages (*N* = 2, 3, … ) corresponded to the described protocol: pumping out the free volume for 1 min, followed by sealing and daily settling. At the end of each stage, the pressure *P* and the value of *W*_exp_ were measured. This degassing procedure allowed us to neglect the decrease in the amount of liquid in the cell, since the foreline pump was switched on for a short time.

In addition, the volume number density of bubstons *n*_*b*_ was measured at pressures P = 1, 3, and 5 atm, created by various gases. For these measurements, the dynamic light scattering (DLS) technique, traditional for such experiments, was used; see, for example, (Chu, 1974; Berne and Pecora, 1990). Visualization of individual nanobubbles was carried out using a laser phase microscope (LPM), see our works (Bunkin et al., 2012; Bunkin et al., 2016; Yurchenko et al., 2016). These setups are detailed below.

### Dynamic Light Scattering

The DLS principle is based on measuring the time-dependent autocorrelation function (ACF) of the intensity of light *I* scattered at a certain angle *θ* in a liquid sample:

$$G^{\left(2\right)}\left(\tau \right)=\langle I\left(t\right)I\left(t+\tau \right)\rangle =\underset{t_{m}\to \infty }{\lim}\frac{1}{t_{m}}\int_{0}^{t_{m}}I\left(t\right)I\left(t+\tau \right)dt,$$ (6)

where $t_{m}$ is the signal acquisition time. For Gaussian radiation statistics, the normalized ACF is expressed by the formula $g^{\left(1\right)}\left(\tau \right)=G^{\left(1\right)}\left(\tau \right)/\langle I\rangle$ (here, ⟨*I*⟩ is the average intensity) is related to the normalized ACF intensity by the Siegert relation$g^{\left(2\right)}\left(\tau \right)=1+a{\left|g^{\left(1\right)}\left(\tau \right)\right|}^{2}$, where *a* is a dimensionless factor that takes into account the spatial coherence of scattered radiation.

For monodisperse particles performing Brownian motion in a liquid, the inverse decay time of the function $g^{\left(1\right)}\left(\tau \right)$ is

$$\tau_{c}^{-1}=Dq^{2}$$ (7)

Here, *D* is the diffusion coefficient of particles in a liquid medium, **q** is the scattering wave vector (corresponding to the Fourier component of spatial fluctuations of the particle position), which is determined from the Bragg relation:

$$q=\left(4\pi n_{0}/\lambda \right)\sin\left(\theta /2\right),$$ (8)

where *n*_0_ is the refractive index of the liquid. Spherical particles with dynamic viscosity $\eta^{\prime }$ and diameter *d*, which move with velocity **u** in a liquid with viscosity *η*, are subject by the Stokes friction force [see, for example, (Landau and Lifshitz, 1987]:

$$F=u\gamma \pi \eta d,\gamma =\frac{2\eta +3\eta^{\prime }}{\eta +\eta^{\prime }}.$$ (9)

In the case of nanobubbles we have γ=4. Note that there often exists an annoying confusion in some articles, devoted to the study of nanobubbles by DLS method: the coefficient *γ* = 6, related to solid particles, is often used for nanobubbles when processing DLS data. The diffusion coefficient of spherical particles, according to the Smoluchowski - Einstein formula, is

$$D=\frac{kT}{\gamma \pi \eta d}$$ (10)

where *T* is the absolute temperature, *k* is the Boltzmann constant. Expansion $g^{\left(1\right)}\left(\tau \right)$ in decaying exponential functions gives a set of correlation times ${\left(\tau_{c}\right)}_{i}=1/D_{i}q^{2}$, where 1 ≤ *i* ≤ *N*, and *N* is the number of fractions of scattering particles with different sizes. Hence, according to Eq. 10, the set of corresponding diameters *d*_*i*_ of particles can be calculated. DLS measurements were carried out using a Zetasizer Nano ZS setup (Malvern, United Kingdom) equipped with a CW helium-neon laser at a wavelength of *λ* = 633 nm (maximum intensity 4 mW), and a temperature controller.

### Laser Phase Microscopy

The LPM method allows one to determine the phase shift profiles *δ* between the reference and object waves, which give an interference pattern on the pixels of the receiving matrix after the object wave passes through a particle in a liquid sample. The quantity *δ* is measured in units of λ/2, where *λ* is the wavelength. If the object wave passes through a spherical particle with diameter *d*, transparent to radiation, then the average value of the optical path difference Δ*h*, measured at the maximum of the interference pattern, is determined by the formula

$$\Delta h=\frac{\delta \cdot \lambda }{2\pi \alpha }=\frac{d\cdot \Delta n}{\alpha },$$ (11)

where *α* is a hardware coefficient, which depends on the particle size. In the approximation of geometric optics, we have α=2. Thus, by measuring Δh, it is possible to distinguish suspended particles with a higher or lower refractive index compared to the surrounding liquid, that is, we can distinguish a gas bubble from a solid particle. In these experiments, we used a laser phase-modulation interference microscope MIM-310 (Amphora Labs, Russia) operating at a wavelength *λ* = 405 nm.

## Experimental Results

**FIGURE 2:**
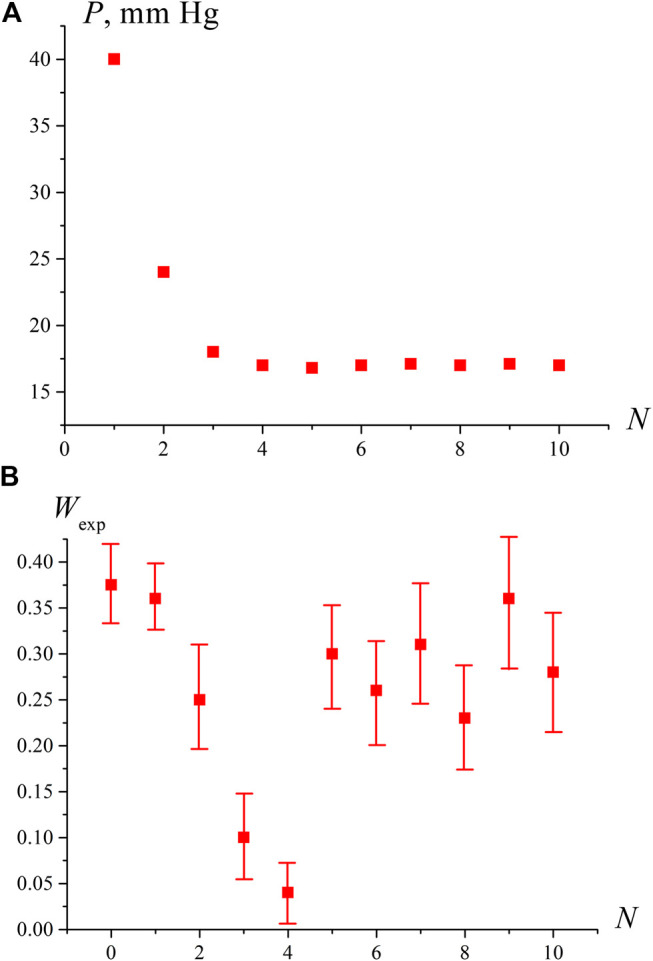
Dependence of pressure P in the free volume of the cell [panel **(A)**] and the probability of optical breakdown *W*_exp_ [panel **(B)**] depending on the degassing number *N*. It can be seen that when the pressure P in the free volume reaches the saturated vapor pressure (*N* ≥ 4), the probability of breakdown increases sharply.

The results of measurements of pressure *P* and the breakdown probability *W*_exp_ are shown in Figures 2A,B as a function of the degassing number *N*. Each experimental point *W*_exp_ corresponds to the averaging over 10 repetitive series, for which the standard deviations were calculated. The number of shots in each series was *N*_0_ = 100. In this experiment, the number of plasma flashes *N*_1_ was measured, and then the value *W*_exp_ = *N*_1_/*N*_o_ was found. The pressure *P* in Figure 2A is the pressure of dissolved gas, which was measured with a manometer; no averaging was performed for pressure measurements. As follows from the graph in Figure 2A, with an increase in *N*, the residual gas pressure *P* decreases, and at *N* = 3, 4 it reaches a stationary level corresponding to the saturated vapor pressure at room temperature *P*_*sat*_ = 17.5 Torr. The fact that the stationary level of residual pressure *P* = *P*_*sat*_ was reached 75–100 h after the start of the degassing procedure is consistent with the estimate of the diffusion time *τ*_*d*_ of dissolved nitrogen from the volume of water with a depth of *h* ≈ 20 mm: *τ*_*d*_ ∼ *h* ^2^/*D*_*g*_ ≈ 85 h (*D*_*g*_ ≈ 1.3⋅10^−5^cm^2^/sec is the diffusivity of nitrogen in water at room temperature).

Establishing a stationary pressure level *P = P*_*sat*_ does not mean that the water is completely free of gas molecules. In such water, with some probability, hydrates of the dissolved gas with an abnormally long lifetime can persist. This assumption is confirmed by the results of measuring the values of *P* and *W*_exp_ presented in Figure2B; the first point on this graph corresponds to water that has not been degassed (*N* = 0). It can be seen from this figure that with an increase in *N*, the probability *W*_exp_ first decreases along with a decrease in pressure *P*, but at *N* = 4, when *P* becomes close to *P*_*sat*_, the probability *W*_exp_ sharply increases and remains high with a further growth of *N*. This is due to the fact that at *P = P*_*sat*_ the liquid is at the boiling point, and vapor bubbles are formed on the remaining bubston nuclei (which in this case will be the centers of optical breakdown), i.e., the volume number density of optical breakdown centers rises significantly. Since radiation at a wavelength *λ* = 1,064 nm is weakly absorbed in water (the absorption coefficient is approximately equal to 0.16 cm^−1^, see, for example, http://www1.lsbu.ac.uk/water/water_vibrational_spectrum.html), we always deal with local heating, and at the boiling point, the volume number density of the boiling/breakdown centers at the areas of laser shooting should increase.

**FIGURE 3:**
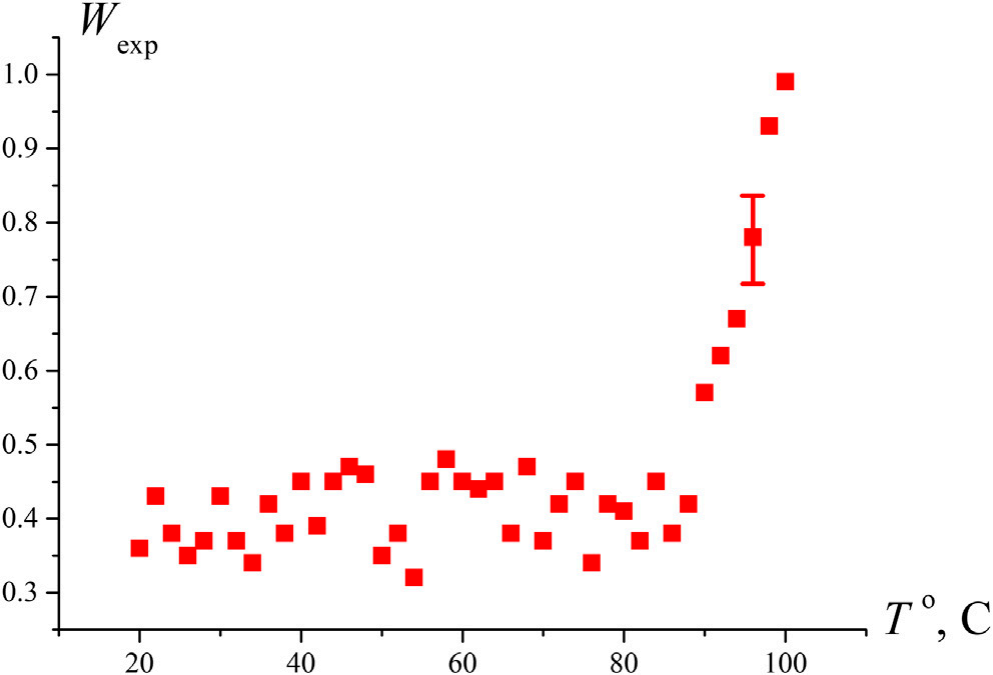
Dependence of the probability of optical breakdown *W*_exp_ vs. the temperature of water in an open cell. It is seen that the density of the breakdown centers increases substantially near the boiling point.

To test the hypothesis that the sharp increase in *W*_*exp*_ at *N* ≥ 4 is due to approaching the boiling point, we measured the probability of optical breakdown *W*_*exp*_ as a function of the temperature in an open cell when the liquid is heated via connecting the cell to a water bath. As seen in Figure 3, the breakdown probability increases sharply at *T* = 97°С, i.e., near the boiling point. Comparing the graphs in Figure 2B and Figure 3, it should be noted that the *W*_*exp*_ data along the ordinate in Figure 2B can be recalculated in accordance with Eq. 6; taking into account *V* ≈ 0.4⋅10^−6^ cm^3^, we can obtain an estimate for the volume number density of bubstons *n*_*b*_. At the same time, in the case of graph in Figure 3, such an estimate will already be incorrect, since at *T* = 97°С the probability of breakdown *W*_exp_≈ 1, i.e., the condition *n*_*b*_ *V* << 1 is violated.

**FIGURE 4:**
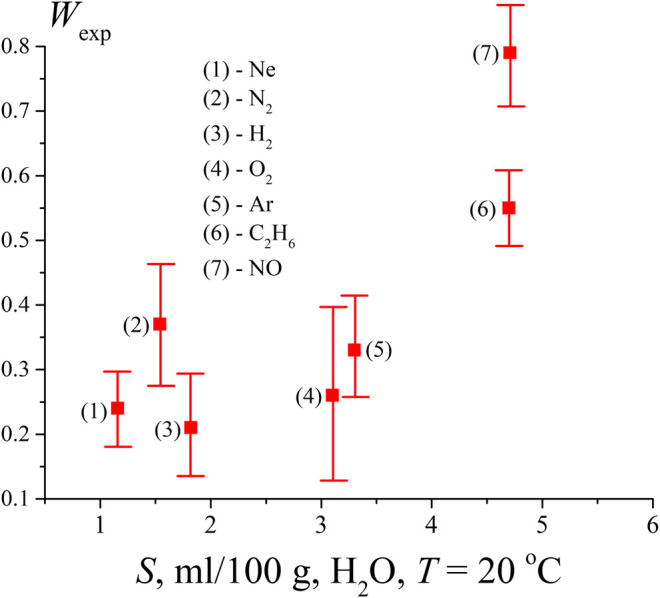
Probability *W*_exp_, measured after saturation of the water sample by gases of various solubility (ml/100 g).

From the results for the pressure *P* ≥ *P*_*sat*_ it would seem that the volume number density of bubstons *n*_*b*_ is proportional to the density $n_{g}^{s}$ of dissolved gas molecules in the liquid, whose hydrates play the role of local inhomogeneities in the liquid medium. However, as shown in *Theoretical Section*, when analyzing the formation of gas cavities (bubston nuclei), it is also necessary to take into account a certain specificity of the local interaction of gas molecules and liquid molecules. According to our model, such specificity may be due to the dipole-dipole interaction between a gas molecule and water dipoles surrounding this molecule. To take this effect into account, the following experiment was carried out. First, the liquid was degassed according to the protocol described above, then 1 atm of test gas was pumped into the free volume of the cell, and the cell was sealed. After 5 days of settling (during this time, obviously, equilibrium is achieved between the gas in the free volume of the cell and the gas dissolved in water), the probability of breakdown *W*_exp_ was measured. In these experiments, neon, nitrogen, hydrogen, oxygen, argon, NO, and C_2_H_6_ were investigated. The results are shown in Figure 4. Since for some gases the breakdown probability *W*_exp_ approaches unity, it was incorrect to recalculate *W*_exp_ using formula (6) to estimate the volume number density of bubstons.

It is clear that the density of bubston nuclei should be controlled by the solubility *S* of a given gas. Since the saturation of the liquid with a dissolved gas occurred at the same initial pressure (1 atm), the effect of different gas solubility should manifest itself in the dependence shown in Figure 4. Additionally, in accordance with our model, the formation of bubston nuclei depends on the efficiency of interaction between a gas molecule and the dipole environment of water molecules, see formula (3); this interaction is controlled by the electronic polarizability of the gas molecules. Table 1 below shows the values of the solubility *S* of the test gases at an external pressure of 1 atm and room temperature (coordinates of the experimental points along the abscissa axis in Figure 4) and their polarizability (data on polarizability are taken from https://cccbdb.nist.gov/pollistx.asp). Note that NO molecules are not only polarizable, but also have a dipole moment μ = 0.16 D.

**TABLE 1:** Solubility and polarizability of gases.

	Ne	N_2_	H_2_	O_2_	Ar	C_2_H_6_	NO
*S*(ml/100 g H_2_O, 20°С)	1.16	1.54	1.82	3.1	3.3	4.7	4.71
*Β* (Å^3^)	0.381	1.710	0.787	1.562	1.664	4.226	1.698

As seen in Figure 4 and Table 1, solubility is not a determining factor for nanobubble formation in a system that is in equilibrium.

As follows from the data in Figure 4, the lowest value of *W*_exp_ (i.e., actually, the volume number density of bubstons *n*_*b*_) is observed for hydrogen. At the same time, hydrogen has approximately the same solubility as nitrogen, and exceeds the solubility of neon, while the polarizability of hydrogen is approximately 2 times higher than the polarizability of neon. Thus, approximately the same *W*_exp_ values for hydrogen and neon, apparently, cannot be explained within the framework of the model outlined in *Theoretical Section*, see formula (3). At the same time, formula (3) is applicable if we compare hydrogen and nitrogen: the polarizability of nitrogen is 2.2 times higher than the polarizability of hydrogen with approximately the same solubility. Note that the results of (Hemmingsen, 1975) can also be interpreted in the context of the efficiency of the Coulomb interaction of gas particles and water molecules. Indeed, in (Hemmingsen, 1975), cavitation was practically absent when water was supersaturated with helium, see Figure 2 of this work. At the same time, the polarizability of helium is the lowest among other gases and equals 0.208 Å^3^, see https://cccbdb.nist.gov/pollistx.asp. Note, however, that surface helium nanobubbles appear under normal conditions on a hydrophobized substrate, see (Van Limbeek and Seddon, 2011).

Furthermore, as follows from Table 1, NO and С_2_Н_6_ have the same solubility, while the ethane polarizability is about 2.5 times higher than that of nitrogen monoxide. At the same time, the volume number density *n*_*b*_ in the case of ethane is lower than that of NO. We attribute this to the fact that the NO molecule has its own dipole moment, i.e., the Coulomb interaction of this molecule with the environment of H_2_O molecules is much more efficient. As noted above, we do not present numerical estimates here, since we do not know the effective dipole moment of the water molecules surrounding the NO molecule (Eq. 3). Finally, according to the data in Table 1, Ar and O_2_ have approximately the same solubility. However, argon has a higher polarizability; in our opinion, this may be due to the higher value of *W*_exp_ in the case of argon. In addition, the chemical reactivity of oxygen in water should be taken into account (for example, with the formation of hydrogen peroxide). Therefore, most likely, the comparison of argon and oxygen in the context of using formula (3) is not entirely correct.

**FIGURE 5:**
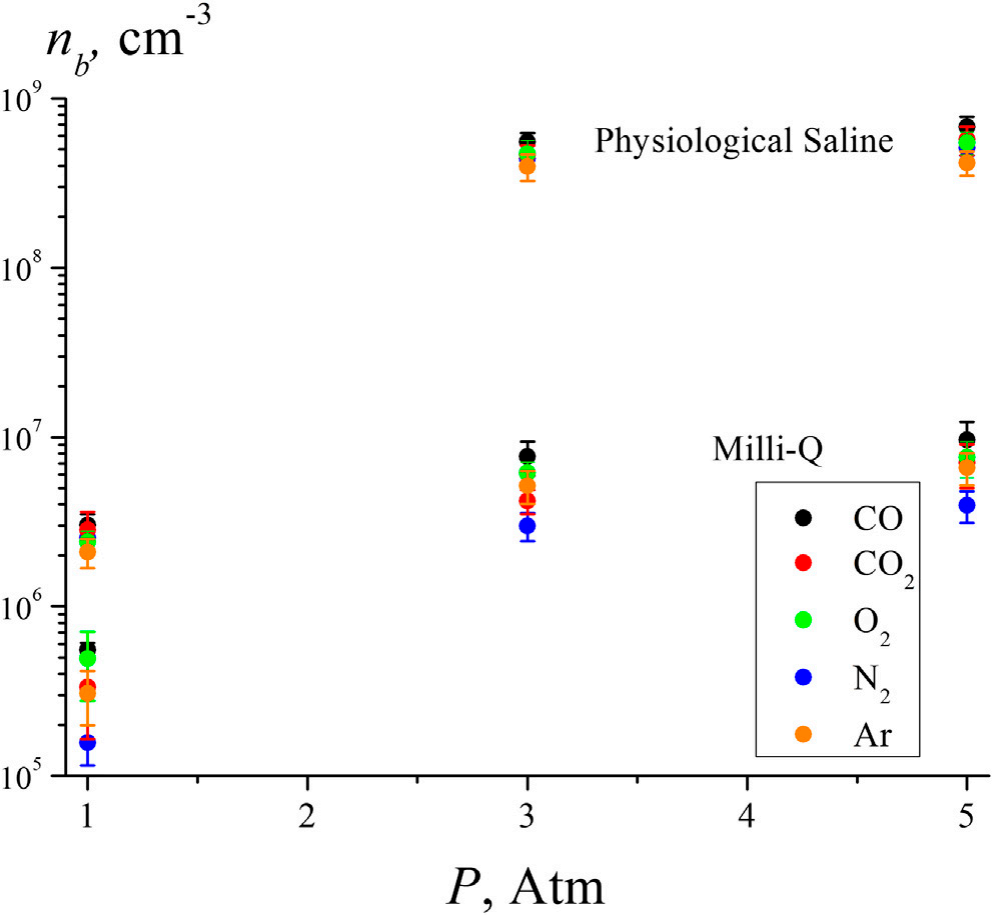
Volume number density of bubstons according to the DLS experiment in water and saline solution for various gases at pressures of 1, 3, and 5 atm.

Figure 5 shows the results of the measurements of the volume number density of bubstons in deionized water (Milli-Q, resistivity 7 MΩ cm, pH = 5.5) and physiological saline solution prepared with Milli-Q water (0.14 M NaCl) under saturation with various gases. Liquid samples with a volume of 200 ml were saturated with the studied gases at pressures of 1, 3, and 5 atm in sealed vessels for 3 days. Liquid samples were preliminarily degassed by boiling under vacuum for 10 min. In this case, a certain amount of liquid evaporated (the quantity of evaporated liquid was not controlled in this experiment), and measurements of the dissolved gas content after boiling were not carried out. Obviously, to measure *n*_*b*_ at external pressures of 3 and 5 atm there was no need to control the gas content of the liquid after degassing. At the same time, the results, obtained with water saturation at 1 atm should not differ significantly from our earlier results of measuring the volume number density of bubstons in water by the DLS method [see (Bunkin et al., 2016), where it was found that *n*_*b*_ ∼ 10^6^ cm^−3^], i.e., the results obtained for different gases saturating a liquid sample at 1 atm can be considered as verification.

After a sharp depressurization, a 1.2 ml sample was taken from the upper layer of the liquid and transferred to a 4.5 ml polystyrene square cuvette with a volume of 10 × 10 × 45 mm^3^ (Sarstedt, Germany) for DLS measurements with a Malvern Zetasizer setup. Each measurement was repeated 5 times; the mean values and standard deviations were determined. Unfortunately, we did not have the opportunity (for technical reasons) to measure the rate of release of gas molecules from bubstons into the liquid bulk for all studied gases.

As shown in our works (Bunkin et al., 2012; Bunkin et al., 2016; Yurchenko et al., 2016), the results of DLS experiments should always be verified in LPM experiments. Indeed, the results of the DLS experiment can be interpreted in favor of bubstons provided that the refractive index of the scatterers found in the DLS experiment corresponds to the refractive index of gas particles (see below).

**FIGURE 6:**
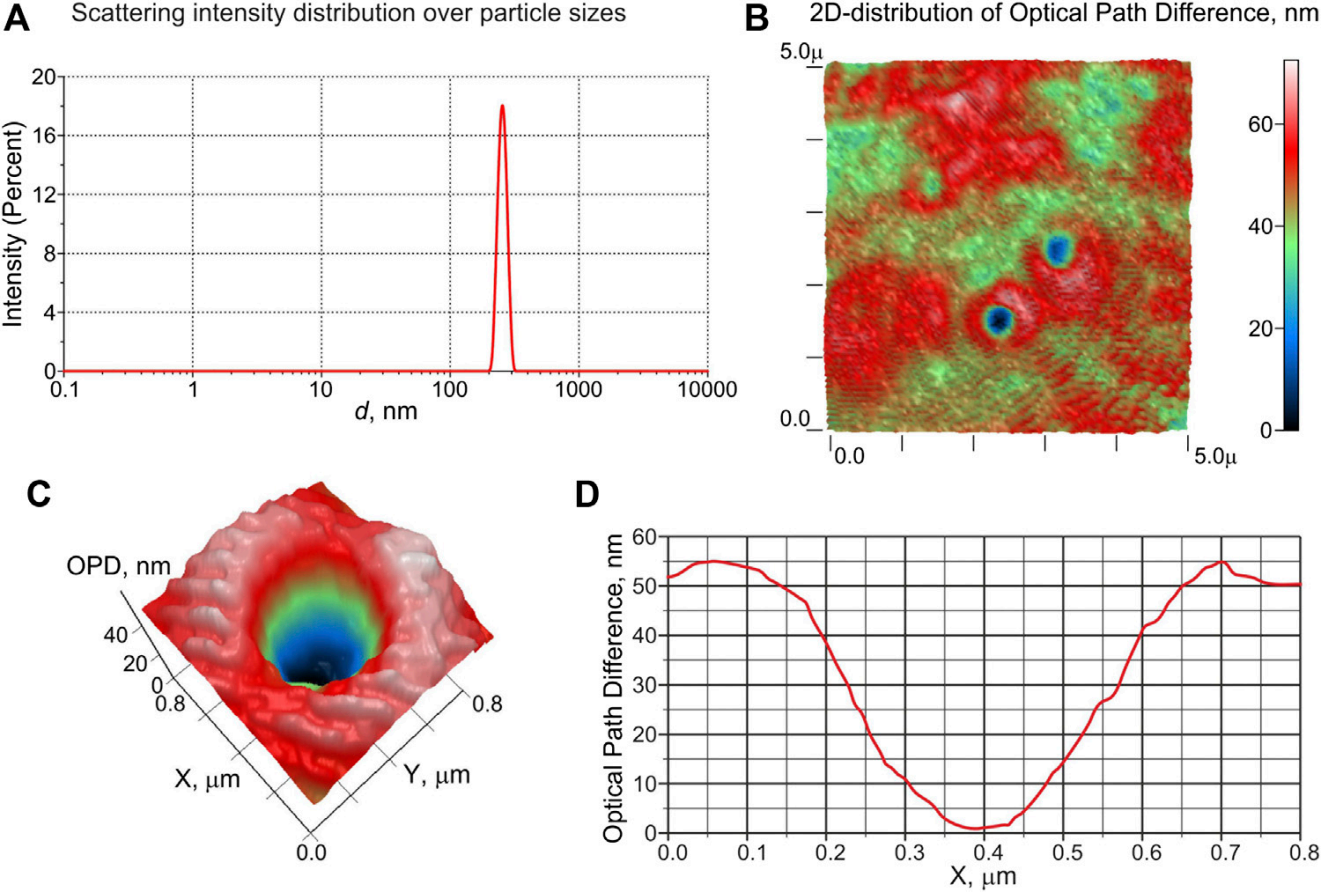
Results of the experiments with DLS and LPM for physiological solutions, saturated with CO_2_ under a pressure of 3 atm. Panel **(A)**: distribution of scattering intensity over scatterer sizes in the DLS experiment. Panel **(B)**: colormap of 2D distribution of the optical path difference (OPD) of a sample area (5 × 5 μm^2^); objects of 300–400 nm in size, for which the OPD distribution has a concave profile, are clearly visible. Panel **(C)**: 2D distribution of OPD in the vicinity of an object with a concave OPD. Panel **(E)**: 1D profile of OPD across the object shown in panel **(C)**; according to the calibration data, the refractive index of this object is *n* ≈ 1.03, i.e., it is gas nanobubble.

A typical histogram of DLS intensity distribution over the scatterer sizes in a saline solution saturated with carbon dioxide at 3 atm is shown in Figure 6 (a). As can be seen, the solution contains particles with an average size of ∼ 250 nm. A 50 μl sample of the same saline solution placed on a glass slide was measured with LPM. In Figure 6B, we exhibit a 2D phase image(color map) of a sample area (5 × 5 μm^2^) with a thickness of about 100 μm; particles with a concave phase profile are clearly visible here. In this figure, the XY-plane corresponds to the area of the liquid sample, and the *Z*-axis is the optical path difference (OPD) between the reference and object waves, measured in nm. Figures 6C,D show 2D distribution of OPD and its 1D profile along the *X*-axis for an individual particle with a concave profile and a size of ∼ 300 nm, respectively. According to formula (11), see the comment in *Laser Phase Microscopy*, the refractive index of the displayed particles is lower than that of the surrounding liquid. An estimate of the refractive index of these particles gives *n* ≈ 1.03, i.e., these particles should be gas nanobubbles formed in the saline after depressurization.

As can be seen from the histogram of the scattering intensity distribution (Figure 6A and phase profiles (Figure 6D), the average particle size observed in the DLS experiments is in good agreement with the size of gas nanobubbles detected in the experiment with phase microscopy. The slight increase in the size of nanobubbles obtained using LPM technique in comparison with the size measured by the DLS apparatus is due to the Brownian motion of the nanobubbles.

Table 2 shows the data for the solubility and electronic polarizability (the data on the polarizability are taken from https://cccbdb.nist.gov/pollistx.asp) of the gases used in this experiment: CO, СО_2_, N_2_, О_2,_ and Ar. Note that the CO molecule is polar: its dipole moment *µ* = 0.11 D. Our special interest in studying the effects of О_2_, СО_2,_ and СО in physiological saline was stimulated by the results of our previous work (Bunkin et al., 2011), where we have observed the effect of bubston formation on the surface of erythrocytes, suspended in saline solution and containing these gases.

**TABLE 2:** Solubility and polarizability of gases.

	CO	CO_2_	N_2_	O_2_	Ar
*S*(ml/100 g H_2_O, 20°С)	2.32	87.8	1.54	3.1	3.3
β (Å^3^)	1.953	2.51	1.710	1.562	1.664

As shown in Table 2, the highest gas content and the highest polarizability are realized for СО_2_. At the same time, the highest number density of bubstons *n*_*b*_ in all liquids and all test pressures is observed for CO. We attribute this to the dipolar structure of CO molecule; note that in the experiment with optical breakdown, the highest bubston density was also observed for the polar gas NO, see Figure 4 Approximately the same density of bubstons is observed for Ar and CO_2_, but for O_2_ the density of bubstons is higher than for Ar and CO_2_ (note that in the experiment with optical breakdown, the probability of breakdown for O_2_ was slightly less than for Ar). In physiological solution, this tendency persists, but there the density of bubstons for all gases is 1 - 2 orders of magnitude higher than in pure water. As follows from our previous works [see (Bunkin et al., 2012; Bunkin et al., 2016; Yurchenko et al., 2016)], this effect is due to the presence of an external ionic impurity. The fact that according to Figure 5, the bubston density for O_2_ in pure water is higher than for all other gases (except for CO) does not agree with our model [*Theoretical Section*, formula (3)]. Indeed, O_2_ has the lowest polarizability among the gases studied in this experiment. In addition, the model based on formula (3) is not applicable in the case of CO_2_: this gas has the highest solubility and the highest polarizability, but the value of *n*_*b*_ in pure water for this gas is less than *n*_*b*_ for O_2_. Apparently, for these gases, chemical reactions in water must be taken into account: in the case of dissolved oxygen, hydrogen peroxide is formed, and in the case of carbon dioxide, carbonic acid. Thus, the model described in *Theoretical Section*, apparently, needs to be refined taking into account possible chemical reactions of gas molecules and water.

The lowest density of bubstons is observed for N_2_; this gas has the lowest solubility among the gases studied in this experiment. We recall that in the experiment with optical breakdown at 1 atm the probabilities of breakdown for N_2_and Ar were approximately the same, but in the DLS experiment, the number density of bubstons in the case of N_2_ is lower than in the case of Ar. At the same time, it is quite surprising that the data on Ar and N_2_ in (Hemmingsen, 1975) devoted to the induction of cavitation in supersaturated gas solutions are very similar. Summarizing this section, we can claim that the data obtained in (Hemmingsen, 1975) are in qualitative agreement with the results presented here. However, it is no point in expecting a complete correlation between the results obtained in our work and those in papers (Hemmingsen, 1975; Rubin and Noyes, 1987; Chen et al., 2014; Chen et al., 2015; Ren et al., 2017; Ren et al., 2020), since in these works, the nucleation of nanobubbles from supersaturated solutions occurs in the absence of nucleation centers, while in our case the bubstons are always present.

## Conclusion

It is shown in this work that, in the pressure range of 1–5 atm, the rate of specific (per unit volume) generation of bubstons *δn*_*b*_/*δt* is determined not only by the volume number densities of dissolved gas molecules $n_{g}^{s}$ and ions *n*_*i*_ see Eq. 2), but also depends on the specificity of the interaction between gas and water molecules. In our experiments, it was found that for the formation of a viable nucleus of a gas nanobubble far from the boiling point, it is necessary for gas molecules to form long-lived complexes with the surrounding water molecules. The interaction of a gas molecule with a dipole environment of water molecules is qualitatively described by formula (3). It follows from this formula that such interaction is most effective for gas molecules with a higher electronic polarizability. All other things being equal, the highest volume number densities of bubstons were obtained for gas molecules with an intrinsic dipole moment.

## Data Availability

The original contributions presented in the study are included in the article/Supplementary Material, further inquiries can be directed to the corresponding author.
